# A Neighborhood-Wide Association Study (NWAS): Example of prostate cancer aggressiveness

**DOI:** 10.1371/journal.pone.0174548

**Published:** 2017-03-27

**Authors:** Shannon M. Lynch, Nandita Mitra, Michelle Ross, Craig Newcomb, Karl Dailey, Tara Jackson, Charnita M. Zeigler-Johnson, Harold Riethman, Charles C. Branas, Timothy R. Rebbeck

**Affiliations:** 1 Fox Chase Cancer Center, Cancer Prevention and Control, Philadelphia, Pennsylvania, United States of America; 2 University of Pennsylvania, Perelman School of Medicine, Philadelphia, Pennsylvania, United States of America; 3 Thomas Jefferson University, Philadelphia, Pennsylvania, United States of America; 4 Old Dominion University, Norfolk, Virginia, United States of America; 5 Columbia University, Mailman School of Public Health, New York, New York, United States of America; 6 Dana Farber Cancer Institute and Harvard TH Chan School of Public Health, Boston, Massachusetts, United States of America; Flinders University, AUSTRALIA

## Abstract

**Purpose:**

Cancer results from complex interactions of multiple variables at the biologic, individual, and social levels. Compared to other levels, social effects that occur geospatially in neighborhoods are not as well-studied, and empiric methods to assess these effects are limited. We propose a novel Neighborhood-Wide Association Study(NWAS), analogous to genome-wide association studies(GWAS), that utilizes high-dimensional computing approaches from biology to comprehensively and empirically identify neighborhood factors associated with disease.

**Methods:**

Pennsylvania Cancer Registry data were linked to U.S. Census data. In a successively more stringent multiphase approach, we evaluated the association between neighborhood (n = 14,663 census variables) and prostate cancer aggressiveness(PCA) with n = 6,416 aggressive (Stage≥3/Gleason grade≥7 cases) vs. n = 70,670 non-aggressive (Stage<3/Gleason grade<7) cases in White men. Analyses accounted for age, year of diagnosis, spatial correlation, and multiple-testing. We used generalized estimating equations in Phase 1 and Bayesian mixed effects models in Phase 2 to calculate odds ratios(OR) and confidence/credible intervals(CI). In Phase 3, principal components analysis grouped correlated variables.

**Results:**

We identified 17 new neighborhood variables associated with PCA. These variables represented income, housing, employment, immigration, access to care, and social support. The top hits or most significant variables related to transportation (OR = 1.05;CI = 1.001–1.09) and poverty (OR = 1.07;CI = 1.01–1.12).

**Conclusions:**

This study introduces the application of high-dimensional, computational methods to large-scale, publically-available geospatial data. Although NWAS requires further testing, it is hypothesis-generating and addresses gaps in geospatial analysis related to empiric assessment. Further, NWAS could have broad implications for many diseases and future precision medicine studies focused on multilevel risk factors of disease.

## Introduction

Cancer likely results from complex interactions of factors at the macro-environmental, individual, and biologic levels[1]. Identifying relevant factors within each level for joint studies is a challenge, particularly at the macro-environmental level, defined here by the neighborhood in which a person lives. Studies of cancer that evaluate neighborhood consider a limited number of variables based on prior knowledge; this affects comparability and consistency across studies and makes etiologic inferences difficult. A lack of empirical assessment is a well-cited limitation of neighborhood studies[2], and empiric approaches from biology could be applied to the macro-environmental level. For example, genome-wide association studies (GWAS) have driven population-based cancer research for the past several years [3]. GWAS use high-throughput, low-cost technology and readily available genome-mapping to evaluate the role of millions of genetic markers for a variety of diseases using an agnostic approach[4]. These approaches are hypothesis-generating, and the clinical implications of GWAS are starting to have translational impact[5].

Applying concepts from GWAS, environmental-wide association studies (EWAS) were subsequently developed to study the effect of exposures at the individual level (e.g., pesticides), and to provide insights for gene-environment interaction studies[6]. However, neighborhood factors have not been comprehensively studied using these approaches. Borrowing concepts from GWAS and EWAS, we propose the neighborhood-wide association study (NWAS) as a novel, empirical approach to evaluate the effect of multiple neighborhood-level exposures on disease outcomes and to address gaps in neighborhood research. The objective of this method is to apply informatics approaches to the study of neighborhood through the systematic identification of neighborhood factors that may be associated with disease phenotypes. With NWAS, we aim to generate hypotheses in order to inform gene-environment studies and potentially more precisely identify neighborhoods at high risk for poor cancer outcomes.

We introduce the NWAS approach and demonstrate how agnostic, high-dimensional data analyses can be used to identify neighborhood characteristics associated with high grade/high stage, aggressive prostate cancer. There are at least two hypotheses that may explain the role of neighborhood in prostate cancer aggressiveness. First, unfavorable neighborhood environments may exert a biological effect on prostate cancer aggressiveness. Neighborhood environment could affect prostate cancer severity under a chronic stress hypothesis, in which residents from disadvantaged neighborhoods experience greater emotional stress and constant “wear and tear” on the body that can affect cancer initiation and progression[7] [8, 9]. Second, unfavorable neighborhood environments may be correlated with factors related to health care access, particularly screening behaviors and practices. Because screening can detect cancer at earlier stages, people living in less favorable neighborhoods may have less access to care that lead to later (i.e., more aggressive) cancers at the time of diagnosis[10–13]. These two hypotheses are not mutually exclusive of one another and could both be acting through neighborhood-level influences. Given few individual-level risk factors for prostate cancer have been identified[14] and only a few studies have investigated neighborhood effects on prostate cancer using *a priori* variable selection approaches[10–13], empiric assessments of the effect of neighborhood on aggressive prostate cancer are warranted.

## Materials and methods

### Study population

Anonymized data from the Pennsylvania (PA) Department of Health Cancer Registry identified prostate cancer patients diagnosed from 1995 to 2005. The registry included variables related to prostate cancer tumor stage and grade, age at diagnosis, year of diagnosis, and race/ethnicity. We focused only on Caucasian prostate cancer cases in this analysis (n = 80,575). Race-specific analyses were also conducted in GWAS to account for population stratification [3, 15]. We excluded cases with a P.O. Box address (n = 112). We also excluded those missing tumor grade or stage (n = 3371), age (n = 2), or year of diagnosis (n = 4). A total of 77,086 men were included in the analysis (S1 File).

### Neighborhood variables

Residential addresses of prostate cancer patients were geocoded at the census tract level and assigned a Federal Information Processing Standard (FIPS) code[16] using Arc GIS software. The FIPS code was linked to the 2000 U.S. Census using Microsoft Visual Studio 2008. Prostate cancer cases were linked to the neighborhood variable values of the census tract in which they live, and cases residing in the same census tract were assumed to have the same neighborhood characteristics.

All 24,634 census tract variables available in the 2000 U.S. Census Summary File 1 (SF1) and Summary File 3 (SF3) were downloaded from Social Explorer (http://www.socialexplorer.com). The SF1 form is distributed to every household in the U.S. SF1 collects demographic data about each person within the housing unit, such as age, gender, and race, as well as general housing information related to occupancy and tenure. The SF3 form is distributed to 5% of all housing units in the U.S and includes more specific questions related to socioeconomic status and physical environment characteristics, such as migration, language ability, disability, veterans status, vehicle availability, kitchen and plumbing facilities [17, 18]. All SF1 and SF3 variables were evaluated for missing data (S1 File; S1 and S2 Figs; S2 and S3 Files). Variables with greater than 10% missingness (n = 8,092) and modal values that comprised over 95% of the data (n = 1,879) were excluded. 14,663 census variables were left for analysis.

### Outcome definition

All incident, White prostate cancer cases among men residing in Pennsylvania from 1995–2005 were included in this study. Incident cases were identified according to ICD-0-3 site and morphology coding. We assumed complete case ascertainment, given medical facilities are required by law to report all diagnosed prostate cancer cases[19] We created a combined “prostate cancer aggressiveness” variable for our primary outcome that was defined by 6,416 cases with a high tumor stage(stage 3 or 4) and high tumor grade(grade 7+), compared to 70, 670 controls(<Stage 3 or <Gleason 7)[20, 21](S1 File).

### Statistical analysis

The NWAS consists of methodologic steps derived from GWAS and EWAS[22] [6, 23]. First, we consider all publically-available Year 2000 U.S. Census variables, which serve as neighborhood “loci”, measured across cases and controls, for associations with prostate cancer aggressiveness after adjustments for multiple comparisons[6]. Second, we account for spatial effects which assume that nearby neighborhoods have similar characteristics[23], an effect that is not consistently accounted for in neighborhood studies and is considered a limitation[2]. Third, we account for linkage disequilibrium statistically and consider the high degree of correlation among census variables [22]. Fourth, dimension reduction techniques were applied across 3 analytical phases, where each phase included progressively more stringent statistical criteria to minimize false positives. All models were adjusted for age at diagnosis and year of diagnosis.

#### Phase 1

The goal of phase 1 of the NWAS analysis was to identify an initial set of neighborhood-level variables associated with unfavorable prostate cancer prognosis, accounting for non-independence of observations within neighborhoods. For each neighborhood variable, a Generalized Estimating Equation (GEE) approach with a logit link function, robust standard error, and assumption of an exchangeable correlation matrix was used to estimate an odds ratio (OR) and 95% confidence interval[24]. P-values were Bonferroni-corrected to account for multiple comparisons[25]; corrected p-values less than 0.05 were considered to be statistically significant. Phase 1 analyses were conducted using SAS 12.0 statistical software.

#### Phase 2

The purpose of the statistical methods in Phase 2 was to further evaluate those variables that reached statistical significance in Phase 1 by accounting for spatial variability in our data. To accomplish this, we specified a Bayesian hierarchical logistic regression model in which we allow for both global and local smoothing using two sets of random effects (see S1 File).

We defined the geographic region as county since each geographic area must include at least 1 case and 1 control. Neighborhood variables were Z-score transformed in order to compare odds ratios from many regressions[6]. To address the large multiple testing problem, our significance threshold is set to 0.05/n for n the number of variables identified in Phase 1, which corresponds to a Bonferroni corrected threshold of 0.05. Significance is determined by the exclusion of 0 in the (100–0.05/n)% credible intervals (CI). Phase 2 analyses were conducted using Integrated Nested Laplace Approximations (INLA) [26] in R statistical software version 3.1.3.

#### Phase 3

The goal of the statistical methods in Phase 3 was to identify variable groups that reflect correlated neighborhood concepts. Paralleling the idea of haplotype blocks (SNPS in high linkage disequilibrium) in GWAS that represent similar gene regions[6], we clustered together significant variables from Phase 2 using principal components analysis[27]. Like post-GWAS fine mapping approaches[28], the most significant variable within each component (i.e., the variable with the tightest credible interval from Phase 2) was considered the best representation of that principal component (i.e. gene region). The most significant variables, or “top hits” were selected from each individual principal component that together explained 90% of the variance. Thus, there were as many top hits as principal components. Phase 3 analyses used STATA/SE 12.0 statistical software. This study utilized existing data sources which allow for waivers of informed consent and was approved by the Institutional Review Board of the University of Pennsylvania under protocol 817734.

## Results

Of the reported 3,135 census tracts in PA in 2000, 3,037 (97%) census tracts are represented in our study sample (S1 File). Aggressive prostate cancer cases were clustered in urban areas, namely Pittsburgh and Philadelphia (S1 File). The average age of the study population was 69.2 (standard deviation (sd): 9.4) and mean year of diagnosis was 2000. The average age of aggressive cases was 69.8 (sd: 10.4) and of nonaggressive cases was 68.8 (sd: 9.0).

**Fig 1:**
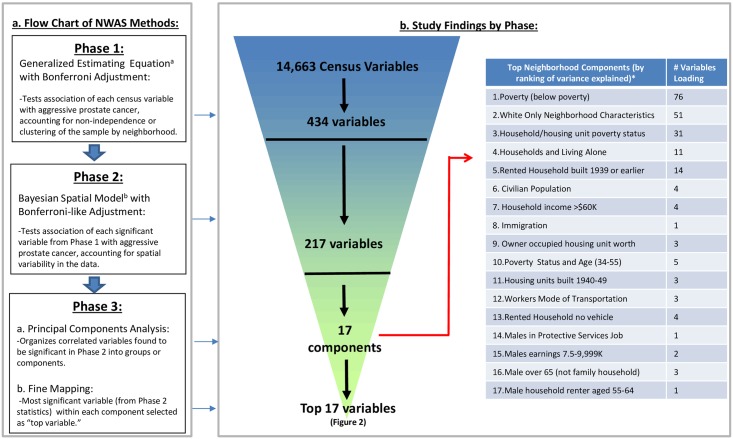
a. Study flow chart of NWAS statistical methods b. Overview of study findings by methodological phase. ^a^ Logit(p) = α+β_i0_x_age_+ β_i1_x_year of diagnosis_ + β_i2_x_neighborhood variable (i, j)_ + ε_ij;_ where i = individual cancer cases; j = census tracts (Phase 1) ^b^ Logit(p) = α+β_i0_x_age_+β_i1_x_year of diagnosis_+β_2_x_neighborhood variable (i, j)_ +V_(j)_ +U_(j)_ where i = individual prostate cancer cases; j = county, V_***(j)***_ are independent non-spatial random effects and U_(j)_ are spatially structured random effects (Phase 2). *These 17 components explain 90% of the variance

Fig 1 summarizes Phases 1–3 study methods (Fig 1a) and findings (Fig 1b). In Phase 1, from 14, 663 variables, we identified 434 unique census variables significantly associated with prostate cancer aggressiveness at Bonferroni-corrected significance levels (S1 and S4 Files). After Phase two, 217 unique variables were still significant at Bonferroni-corrected credible intervals (S5 File). The average amount of residual variability in aggressive prostate cancer risk across the 217 Bayesian models in Phase 2 was 34% (range: 14%-50%), which is considered large. In Phase 3, 17 uncorrelated principal components were identified from these 217 neighborhood variables. Components 1–8 explain 80% of the variance, and related to poverty (Component 1), white only neighborhood characteristics (Component 2), household/ housing unit poverty status (Component 3), households and living alone (component 4), rented houses built before 1939 (Component 5), civilian population (Component 6), household income above $60K (Component 7), and immigration (Component 8) (Fig 1b). 76 of 217 Phase 2 variables loaded on Component 1 and 51 loaded on Component 2 after the Phase 3 principal components analysis. The top 10% of significant variables from Phase 2 loaded on Component 1 (S5 File).

**Fig 2:**
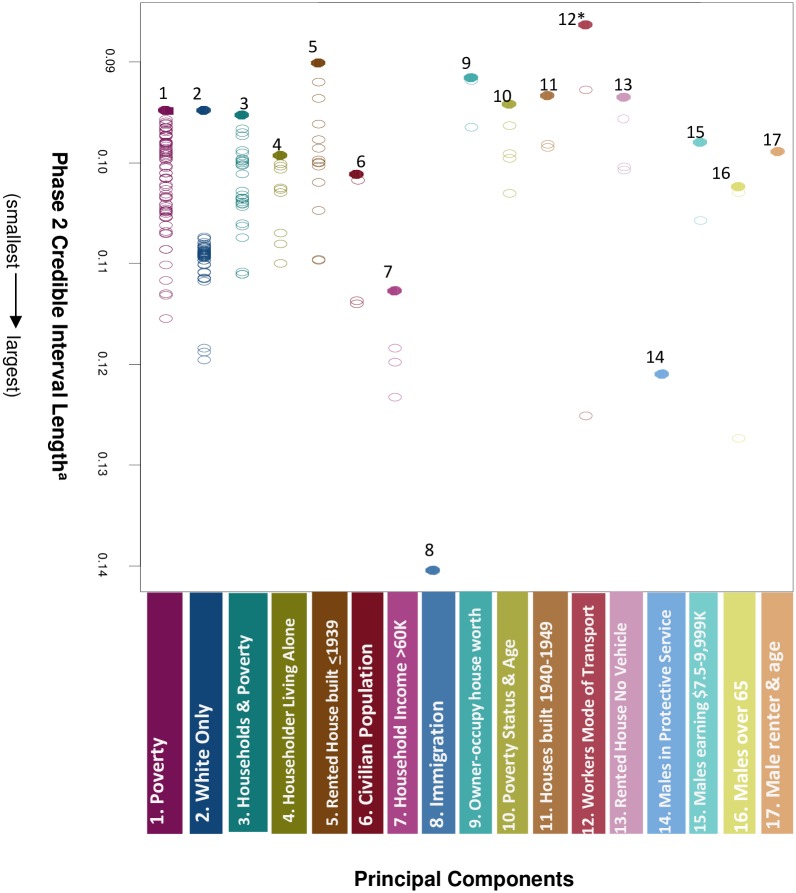
Phase 3-Principal components and fine mapping analysis to identify top hits. Dots represent single neighborhood variables from Phase 2 (n = 217 total dots). Open dots are color-coded to their respective component (from Phase 3-Principal Components analysis). Closed-colored dots represent the most significant variable within each component (Phase 3-Fine Mapping) and corresponding statistics are provided by component number in Fig 3. *Top hit based on statistical significance from Phase 2 data. ^a^ Statistical significance determined by Bonferroni-corrected confidence intervals from the Phase 2 Bayesian model, i.e. smaller credible interval length indicates greater statistical significance.

**Fig 3:**
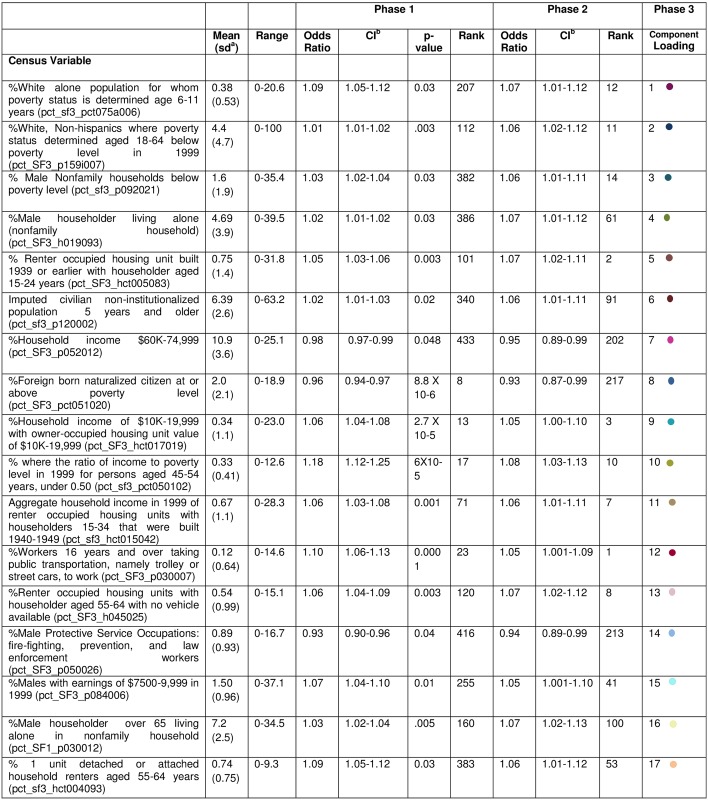
Summary of neighborhood variable “top hits” associated with aggressive prostate cancer by phase. ^a^Standard deviation (sd); ^b^Confidence or Credible Interval (CI).

The top 17 most significant variables within each of the 17 principal components are presented in Figs 2 and 3. The most significant variable from Component 1 represented Non-Hispanic Whites aged 6–11 for whom poverty status was determined (OR = 1.07, CI = 1.01–1.12). Seven of the top 17 hits or variables from Components 1–3, 7, 9–10, 15 were related to socioeconomic status. One top hit related to employment, specifically male protective service occupations such as fire-fighting and law enforcement (OR = 0.94, CI = 0.89–0.99), and one related to immigration status (OR = 0.93, CI = 0.87–0.99). Two were associated with physical environment (aggregate income of occupied, rented housing units built 1940–1949 with a householder aged 15–34 (OR = 1.06, CI = 1.01–1.11) and percent (%) renter occupied housing units built 1939 or earlier with householder aged 15–24 years (OR = 1.07, CI = 1.02–1.11). Two variables related to social support (%male householder living alone (OR = 1.06, CI = 1.01–1.11) and %male householder over 65 living alone in nonfamily household (OR = 1.07, CI = 1.02–1.13)). The top hit (most significant variable from Phase 2) was %workers >16 years taking trolley or street car public transportation to work (OR = 1.05, CI = 1.001–1.09).

## Discussion

We used a novel NWAS to assess the association of 14,663 neighborhood variables with prostate cancer aggressiveness from the PA Cancer Registry. Through a series of progressively more stringent phases, model adjustments, and dimension reduction techniques, we identified the top 17 neighborhood variables associated with aggressive prostate cancer. These findings confirm some previous associations, but also provide new insights into the role of neighborhood in prostate cancer and suggest the potential value of NWAS to inform public health interventions and multilevel studies.

Previous studies of neighborhood and prostate cancer suggest that neighborhoods with poor socioeconomic (SES) circumstances are related to high-grade prostate cancer[29], independent of individual-level exposures[12, 20]. In these studies, SES was measured with deprivation scores and single, *a priori* selected U.S. census variables related to education, income, poverty, housing[30] and employment (S1 File). Our findings support that neighborhood income and poverty (Components 1, 2, 3, 7, 8, 9, 10, 15), employment (Component 14) and housing (Components 3, 4, 5, 9, 11, 13, 16, 17) relate to prostate cancer aggressiveness. However, neighborhood education was not an important determinant of aggressive prostate cancer here, suggesting education could be a confounder rather than a main effect in neighborhood studies, but more study is warranted.

Immigration (Component 9; %foreign-born naturalized citizens at or above poverty) was a significant NWAS finding. Studies of neighborhoods with higher rates of foreign-born immigrants have shown associations with decreased risk for cancer[31]. Even if individuals are diagnosed with late-stage prostate cancer, survival is improved for those who live in high ethnically homogeneous enclaves, suggesting the strong role social support, alone and in conjunction with poverty, may play in prostate cancer progression[31, 32].

The top hit in this analysis related to taking public transportation to work. This variable, as well as not owning a vehicle, relate more to urban, as compared to rural settings, and are also often used as surrogates for access to medical care[33, 34]. Access to care is often cited as a cause of disparity in prostate cancer treatment[34] and survival[33] across both urban and rural settings. Higher cancer incidence and mortality rates are noted in more urban settings, and cases arising from rural environments often are diagnosed at later disease stages[35]. Thus, NWAS findings are plausible and consistent with previously identified sociodemographic domains[2, 36].

From a methodologic standpoint, NWAS provides a new, agnostic approach to neighborhood and contextual variable selection[2]. In the past, one study might define poverty as proportion of households below poverty, another as percentage receiving public assistance. This inconsistency in the choice of neighborhood or contextual variables has limited the ability to make etiologic inferences across studies[27]. Further, previous neighborhood studies often select census variables that represent fewer socioeconomic parameters, for instance, % population below poverty[17][10]. Our NWAS identified more complex, joint effect variables that combined race, age, and poverty information with household or renter status. These more complex variables could provide insights into disease etiology and suggest that interactions may exist among demographic domains that are often considered individually in current neighborhood studies[10]. For example, percentage of male nonfamily householders living alone AND percentage of male nonfamily householders living alone over 65 appear to represent similar social concepts. However, NWAS separated these variables into two different components. Variables related to single resident households are used as markers of social support[37, 38], and it is possible that they could represent separate or potentially dynamic changes in the role of social support across the lifespan. Thus, given the specificity of NWAS top hits, it is possible they could be used alone or in combination, in future multilevel investigations and to more precisely identify and target geographic areas that are associated with one or more unfavorable NWAS characteristics for disease interventions. However, NWAS is a new methodologic approach and the etiologic significance of the NWAS hits would need to be investigated within those neighborhoods that exhibit these unfavorable characteristics. Further, the utility of NWAS findings for neighborhood risk assessments will need to be determined through comparisons with existing variable selection methods in future studies.

While NWAS methods can be extended in a variety of ways, the current formulation described here has limitations. Area-level data analyses assume individuals residing within the same geographic area experience similar circumstances. In reality, non-residential experience (e.g., work) and individual-level characteristics (e.g., biology, behavior, risk factors) also impact health states. Thus, future NWAS should be conducted in study populations that can adjust for or directly study individual-level SES factors[29]. In addition, standardized data processing, aggregation methods, and geographic boundaries used in administrative datasets can suffer from systematic reporting bias and missingness [6, 16]. Based on our missing data assessments, bias is likely non-differential (S1 File), but future NWAS studies should investigate missingness using both spatial autocorrelation and imputation techniques[39], as well as evaluate the effects of aggregation and geospatial boundary selection using interpolation and point-based, boundary-free approaches [40, 41] [42, 43].

The NWAS approach described here features many of the methodologic requirements previously proposed for GWAS or EWAS studies[23]. A hallmark of GWAS has been replication of discovery findings in comparable study populations. The NWAS presented here focuses on discovery and minimization of false positives through statistical adjustments, without a separate replication population. Under certain circumstances, a single discovery phase[44] and other biologic or functional-based approaches may be favored over statistical replication[45]. For example, the frequency or percentages of census variables may vary by geography, which can bias estimates of association. Interactions between variables (as indicated by the more complex, joint effect variables in the NWAS) are also likely to vary by geography[45]. While comparisons across geographical areas may be undertaken, use of independent datasets to validate findings may mask real differences between these geographies, and may not be appropriate in the NWAS setting. This is a topic that requires further exploration.

This NWAS study demonstrates that high-dimensional data analysis can be applied to large, publically-available datasets and can yield biologically plausible results. This is the first study to systematically, agnostically, and comprehensively evaluate the role of neighborhood-level factors in prostate cancer using “big data” methods. Although NWAS approaches should be tested in other study populations, NWAS addresses methodological limitations in current neighborhood studies, while capitalizing on methodologic approaches used for precision medicine[46] [47]. Further, coupling an NWAS approach with individual-level risk factor information could have implications for multilevel, health disparity studies, as well as precision public health initiatives aimed at identifying and targeting geographic areas in need of intervention efforts across disease sites.

## Supporting information

S1 Fig Year 2000 SF1 variable missingness overview for the state of Pennsylvania. (TIF)

S2 Fig Year 2000 SF3 variable missingness overview for the state of Pennsylvania. (TIF)

S1 File Methods and data analysis. Data CleaningData MappingNeighborhood-wide Association Study (NWAS) Methods DetailTable. Examples of Neighborhood Methods used in Prostate Cancer Research
(DOCX)

S2 File Summary of year 2000 U.S. census SF1-Pennsylvania state prostate cancer registry join. (XLSX)

S3 File Summary of year 2000 U.S. census SF3-Pennsylvania state prostate cancer registry join. (XLSX)

S4 File Phase 1 results. (XLSX)

S5 File Phases 2 and 3 results. (XLSX)
