# Supplementary figures and images for: A Neighborhood-Wide Association Study (NWAS): Example of prostate cancer aggressiveness
Source: PLoS One. 2017 Mar 27;12(3):e0174548. doi: 10.1371/journal.pone.0174548 (PMC5367705; doi:10.1371/journal.pone.0174548)

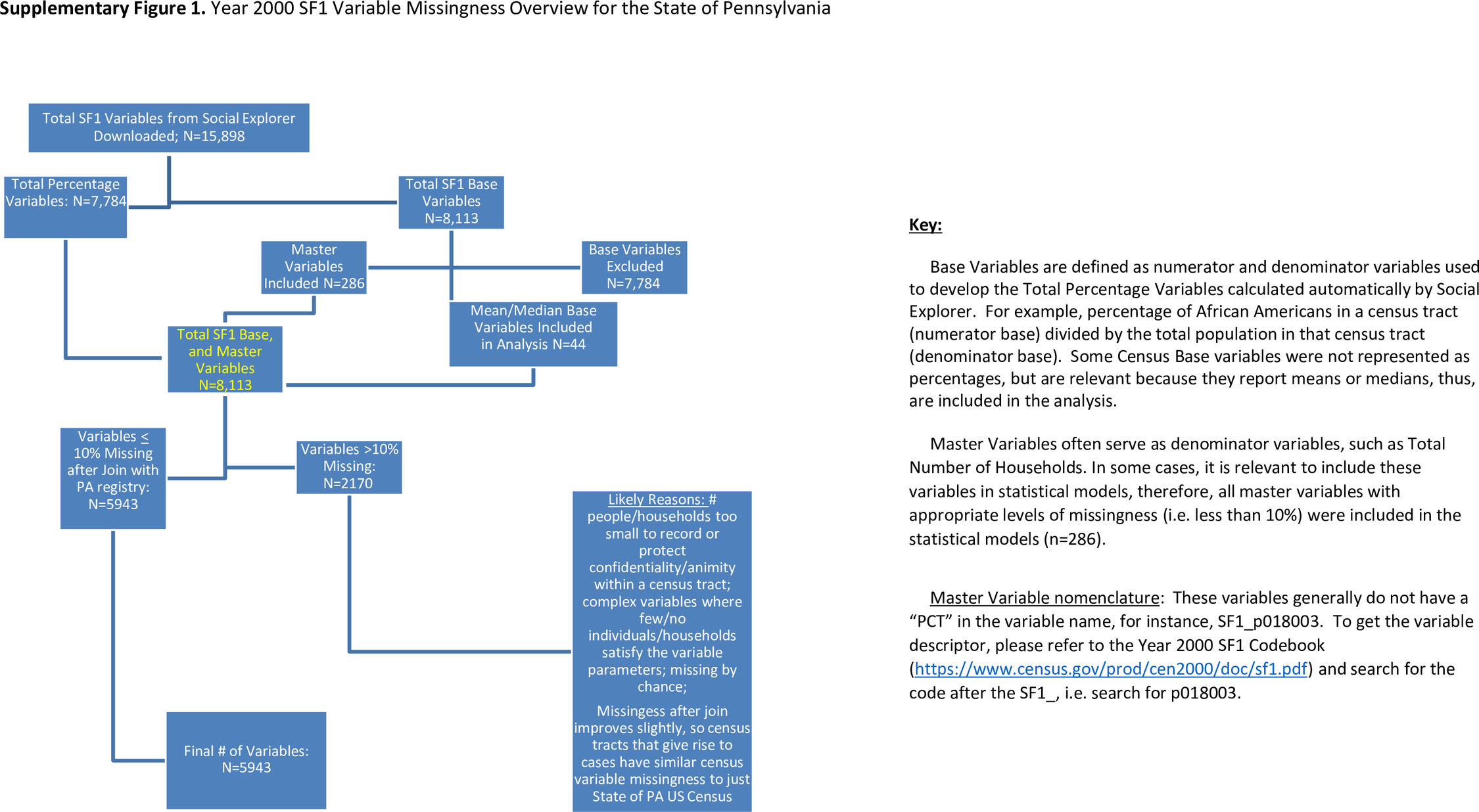

Supplement: S1 Fig — (TIF) [file pone.0174548.s001.tif]

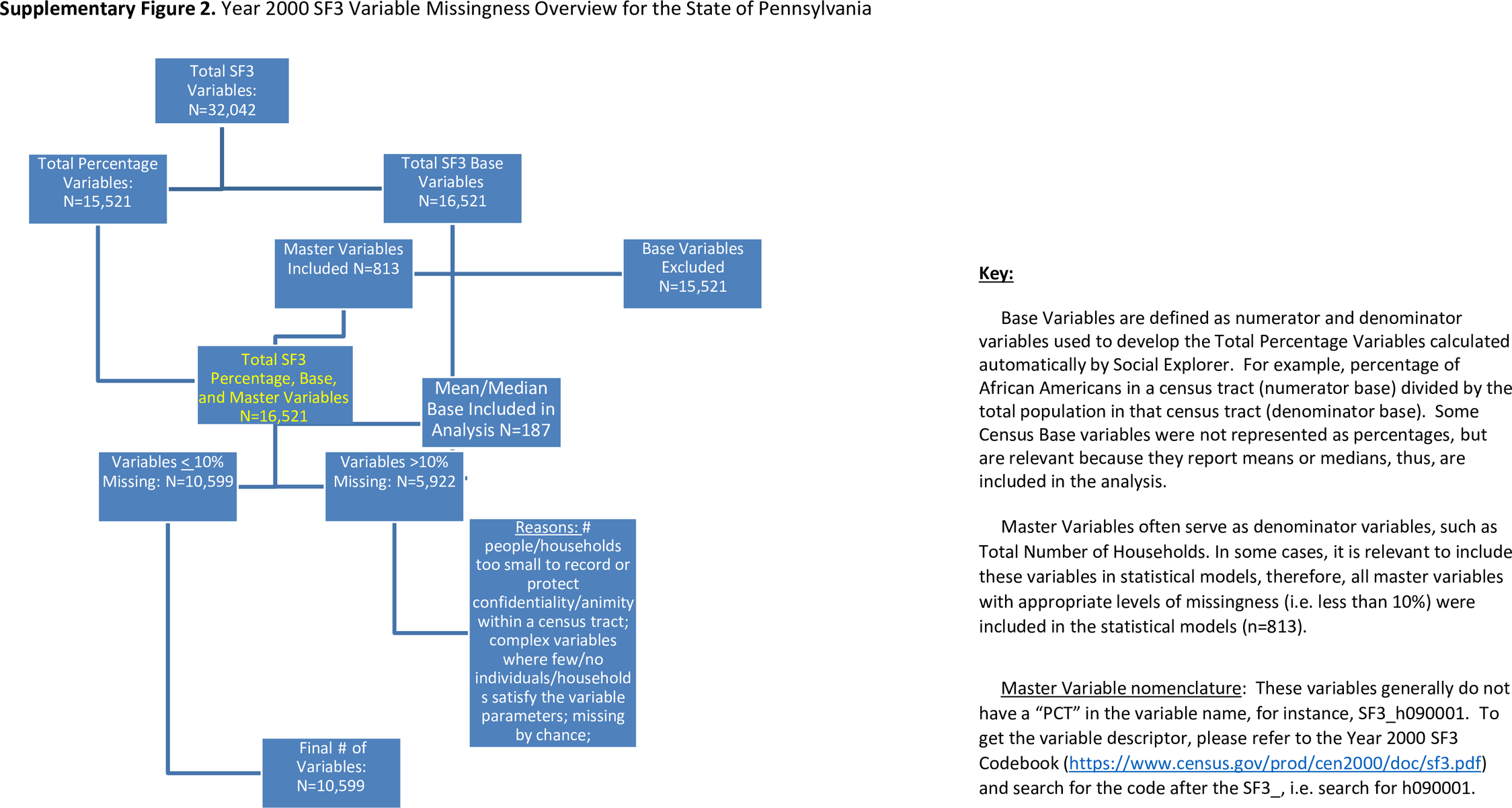

Supplement: S2 Fig — (TIF) [file pone.0174548.s002.tif]
