# Supplementary material for: A Neighborhood-Wide Association Study (NWAS): Example of prostate cancer aggressiveness
Source: PLoS One. 2017 Mar 27;12(3):e0174548. doi: 10.1371/journal.pone.0174548 (PMC5367705; doi:10.1371/journal.pone.0174548)
Supplement: S1 File — Data CleaningData MappingNeighborhood-wide Association Study (NWAS) Methods DetailTable. Examples of Neighborhood Methods used in Prostate Cancer Research (DOCX) [file pone.0174548.s003.docx]

**NWAS Supplementary Material**

**Supplement 1-Methods and Data Analysis**

1. **Data Cleaning:** Characterization of Data Missingness by Outcome and Neighborhood Variables

**Prostate Cancer Outcome.** Overall, there were 93,308 incident cases of prostate cancer between 1995-2005 in the State of Pennsylvania. We focused this analysis on Caucasians only (prostate cancer cases=80,575). 112 prostate cancer cases were dropped because their home address was a non-descript P.O. Box address, 6 more were dropped because of missing age and date of diagnosis, leaving 80,457 cases. The main outcome variable, prostate cancer aggressiveness, is a combination variable comprised of tumor stage and grade data. Tumor stage and grade were determined by Surveillance, Epidemiology, and End Results (SEER) coding criteria for stage and histology variables, respectively[1, 2]. Subjects with low stage prostate cancer (Stage 1 and 2) were defined by SEER Stages 0 and 1; subjects with high-stage (Stages 3 and 4) were defined by SEER Stages >2[1, 3]. Low tumor grade (Gleason score of 6 or below) and high-grade (Gleason score of 7 or greater) prostate cancer was determined from the SEER 6^th^ digit coding for histology and differentiation[4]. Sixth digit diagnosis codes that were equal to 6 or 9 were excluded because grade or differentiation could be not determined, was not stated, or was not applicable[4]. The stage variable is never missing, and the grade variable is missing for 3,371 prostate cancer patients(4.2% of total cases). Thus, we had 77, 086 Caucasian cases left for an analysis with our primary outcome variable; 6, 416 were classified as aggressive cancer and 70,670 cases were classified as “non-aggressive.”

**Neighborhood variables.** Selection of Year 2000 U.S. Census variables for inclusion in this analysis from SF1 (short form; entire population completes) and SF3 (long form; subset of the population completes) is summarized in Supplementary Figures 1 and 2 (S1 Fig.; S2 Fig.), respectively. We assessed percent missingness for each census or neighborhood variable from the 2000 US Census SF1(n=8113) and SF3 forms(n=15,521) before and after the join that linked the cancer registry to the US census data. Percentage available for each variable is reported in Supplement 2 (S2 File) for SF1 and Supplement 3 (S3 File) for SF3. Final variables included in the NWAS analysis (SF1 n=5,943; SF3 n=10,599) were based on having less than 10% missing data. Using Supplement 2 as an example, for each census variable, % difference between the census data and the join of the census with the registry data was not more than 3-6.5%. Thus, census or neighborhood variable missingness did not appear to be majorly affected by the incorporation of case status after joining the 2000 U.S. Census data SF1 and SF3 forms to the Pennsylvania State Prostate Cancer Registry (1995-2005).

**Census Tract Level.** Individual prostate cancer cases were linked to Year 2000 Census SF1 and SF3 forms at the census tract level. Of the reported 3, 135 census tracts in the entire State of Pennsylvania in Year 2000, 3,037 census tracts are represented in our Caucasian population. Thus, 97% of PA census tracts are covered in this analysis. On average, there were 2 cases of aggressive prostate cancer compared to 23 controls with non-aggressive prostate cancer in each census tract. We also assessed case status by census block group, but there were <1 cases on average and 8 controls in each census block group. Prostate cancer grade missingness did not appear systematic by census tract (in the grade variable, 3,033 census were represented). Thus, missingness is likely at random.

**Statistical Analysis.** Using the joined (combined registry and census) final dataset, 1346 (SF1=748; SF3=598) neighborhood variables(8% of the final analytic set) would have been excluded based on census only missingness, but were included in the analysis because missingness improved to <10% after the join. After running Phase 1, only 1 of these variables were included in the top 517 hits, and none of these variables were in the top hits after Phase 2, thus findings did not change by this inclusion criteria.

1. **Data Mapping.** Distribution of Aggressive Prostate Cancer in the State of Pennsylvania 1995-2005: A. All Prostate Cancer Cases by Census Tract; B. Aggressive Prostate Cancer Cases by Census Tract
2. **Neighborhood-Wide Association Study(NWAS) Methods Detail**

*NWAS Phase 1*: To be fully inclusive, each neighborhood variable was included in Phase 1 and a Generalized Estimating Equation (GEE) approach with a logit link function, robust standard error, and assumption of an exchangeable correlation matrix was used to estimate odds ratios [5]:

*Logit(p)=* *α+β_i0_x_age_+ β_i1_x_year of diagnosis_ + β_i2_x_neighborhood variable (i, j)_  + ε_ij;_ (Eq. 1),*

where i= individual prostate cancer cases; j=census tracts

After Bonferroni-adjustment, we identified 517 variables from 14,663 that were significantly related to aggressive prostate cancer. Because the census does assign different variable names to similar concepts (i.e. both SF3_h080001 and SF3_h090001 represent owner-occupied housing units), prior to Phase 2, we excluded overlapping census variables based on correlation coefficients of 1.0 and similar variable descriptor names, leaving us with 434 significant variables for Phase 2. Significant findings were similar when excluding overlapping variables prior to Phase 1(S4 file).

*NWAS Phase 2:* For each significant variable from Phase 1, *w*e specified a Bayesian hierarchical logistic regression model in which we allow for both global and local smoothing using two sets of random effects:

*Logit(p)=* *α+β_i0_x_age_+ β_i1_x_year of diagnosis_ + β_2_x_neighborhood variable (i, j)_  + V_(j)_ + U_(j)_ (Eq. 2)*

where i= individual prostate cancer cases; j=county, V(j) are independent non-spatial random effects and U_(j)_ are spatially structured random effects. We model the spatial random effects using an intrinsic conditional auto-regressive (ICAR) prior[6]:

$$U_{j}|U_{k}, k\in\delta_{j} \sim N(\bar{U_{j}}, \omega_{U}^{2}/m_{j}),$$

where $\delta_{j}$ is the set of neighbors of county *j*, *m_j_* is the number of neighbors, $\bar{U_{j}}$ is the mean of the spatial random effects of the neighbors, and $\omega_{U}^{2}$ is the conditional variance whose magnitude determines the amount of spatial variation[6].

This model imposes smoothing by assuming that the spatial effect in a specified geographic region is similar to the mean of the spatial effects in near-by regions, with the strength of the similarity determined by the number of neighbors (i.e., counties with more neighbors will have stronger similarities imposed). We define geographic regions *j* and *k* to be neighbors if they share a common boundary. Under this model, we must assign distributions to $\sigma_{V}^{2}$ and $\omega_{U}^{2}$. We specify a Gamma(0.05,0.001) prior distribution on $\tau_{V}=\sigma_{V}^{-2}$ and $\tau_{U}=\omega_{U}^{-2}$. After Phase two, 217 variables were still significant for Phase 3 analysis (S5 file).

*NWAS* *Phase 3:* In principal components analysis, unique variables were considered ( S5 files). For unique variables that loaded strongly(>0.30; [7-9]) on more than one component, the magnitude of the correlation coefficient for each component, as well as the variable description, were used to determine the most appropriate component placement. Precedent was given first to the magnitude of the correlation coefficient. 17 principal components were identified, with the most significant variable from each component serving as the study “top hits”.

1. **Table 1. Examples of Neighborhood Methods used in Prostate Cancer Research**

| **Study (data source)** | **Neighborhood variables** | **Outcome** | **Results** | **Ref** |
| --- | --- | --- | --- | --- |
| **Method: Neighborhood Indices** | | | | |
| Hellenthal et al. 2010 (CA Cancer Registry) | Principal components  analysis used to create an SES score(1-5, 5 being the highest), including median household  income, education level, proportion below 200% poverty,  and median house value. | PCa Treatment/Survival | Men of lower SES are less likely to undergo radical prostatectomy (RP) or radiation (XRT) for the management of localized prostate cancer. After RP or XRT, men of lower SES have a decreased cancer-specific survival compared with men of higher SES. | [10] |
| Zeigler-Johnson et al. 2011 (PA Cancer Registry) | 1. Analyzed individual SES variables from Census. 2. Calculated a deprivation index (-1 to 1, with 1 being the highest deprivation index) using a principle components analysis (PCA), including:  (1) % of households with income <$30,000/year  (2) % poverty;  (3) % households on public assistance  (4) % female head of household with dependent children  (5) % households with no car. | Prostate cancer | The highest quartile of neighborhood deprivation was also associated with high Gleason score. For both Caucasians and African-Americans, the highest quartile of neighborhood deprivation was associated with high Gleason score at diagnosis (OR=1.27, 95% CI=1.11-1.44; OR=1.61, 95% CI=1.15-2.25, respectively.) Using a neighborhood deprivation index, associations between prostate cancer severity and neighborhood deprivation across ethnic groups was observed. | [11] |
| Cheng et al. 2009 (CA Study) | Principal component analysis to develop single SES index from seven census-based indicator variables of SES: 1. mean years of education; 2. median household income; 3. percent living 200% below poverty level;  4.percent blue-collar workers; 5. percent older than 16 years in workforce without job;  6. median rent;  7. median house value . This index was used to assign a standardized score to each census block group, which was then categorized into quintile levels. | Prostate Cancer risk and mortality | Higher levels of SES were associated with lower mortality rates of prostate cancer deaths (SES Q1 vs. Q5: RR = 0.88; 95% CI: 0.92–0.94). African-Americans had a twofold to fivefold increased risk of prostate cancer deaths in comparison to non-Hispanic Whites across all levels of SES. | [12] |
| Lyratzopoulous et al. 2010 (United Kingdom) | The United Kingdom 2004 Indices of Deprivation. | Prostate Cancer Treatment/Survival | After a diagnosis of prostate cancer, men from lower socioeconomic groups were substantially less likely to be treated with radical surgery or radiotherapy. The causes and impact on survival of such differences remain uncertain. | [13] |
| Byers et al. 2008 (NPCR POC Study) | Both education and income were classified into 2 Levels (<25% vs 25% of adults aged 25 years with less than a high school education and <20% vs 20% of households with incomes below the Federal Poverty Level). Each patient was then classified as living in a census tract with neither low education nor low income (65% of cases), with only 1 of those indicators of low SES (20% of cases), or with both of those indicators (15% of cases). | Advanced Prostate, Breast, colon cancer | Low SES was associated with more advanced disease stage and with less aggressive treatment for all 3 cancers. | [14] |
| Schymura et al. 2010  (CDC-NPCR PoC1) | Records were linked by census tract. The following variables were analyzed individually:  1. poverty (<20% versus 20%+ of residents below the 2000 poverty level);  2. education (<25% versus 25%+ of residents age twenty-five and over with less than a high school education); 3.working class status (<66% versus 66%+ working class occupations);  4. urban-rural residence (totally urban, totally rural, urban-rural mix, or unknown). | Prostate Cancer survival | No neighborhood variables were associated with survival from localized prostate cancer  State of residence was a significant predictor of treatment type and overall survival. | [15] |
| Marlow et al. 2010 (national cancer database) | Socioeconomic status was classified using the median household income and proportion of population with a high school diploma from patient's ZIP code of residence. | Advanced Prostate Cancer | Patients residing in areas with lower socioeconomic characteristics have significantly increased odds of advanced PCa. | [16] |

REFERENCES:

1. *SEER Program Coding and Staging Manual 2000*, E. National Cancer Institute. Surveillance, and Endpoints Research (SEER). Editor. 2000: Bethesda, MD.

2. SEER., *SEER Training Modules*, in *Prostate Cancer* N.I.o.H. U.S. Department of Health and Human Services, National Cancer Institute, Editor. 2014: Bethesda, MD.

3. Zeigler-Johnson, C., Tierney, A., Rebbeck, T.R., Rundle, A., *Prostate Cancer Severity Associations with Neighborhood Deprivation.* Prostate Cancer 2011.

4. *Rule G* SEER Training Modules 2012 October 30, 2013]; Available from: <http://training.seer.cancer.gov/coding/guidelines/rule_g.html>.

5. Hubbard, A.E., et al., *To GEE or Not to GEE: Comparing Population Average and Mixed Models for Estimating the Associations Between Neighborhood Risk Factors and Health.* Epidemiology, 2010. **21**(4): p. 467-474 10.1097/EDE.0b013e3181caeb90.

6. Besag, J., York, J., Mollie, A., *Bayesian Image Restoration, with Two Applications in Spatial Statistics.* Ann Inst Statist Math, 1991. **43**(1): p. 1-59.

7. Messer, L., . Laraia, B., Kaufman, J., Eyster, J., Holzman, C., Culhane, J., et al. , *The development of a standard neighborhood deprivation index. .* Journal of Urban Health, 2006. **83**(6): p. 1041-1062.

8. Tabachnick, B.G., Fidell, L.S., *Chapter 13: Principal Components and Factor Analysis. Using Multivariate Statistics. 3.* Northridge, California: California State University, Harper Collins College, 1996. **635-708**.

9. *Principal Components Analysis*. <http://www.unt.edu/rss/class/mike/6810/Principal%20Components%20Analysis.pdf> University of Texas (accessed 2015).

10. Hellenthal N.J., e.a., *Men of Higher Socioeconomic Status Have Improved Outcomes After Radical Prostatectomy for Localized Prostate Cancer.* Urology, 2010. **76**(6): p. 1409-1413.

11. *Prostate Cancer Severity Associations with Neighborhood Deprivation.* Prostate Cancer, 2011. **2011**.

12. Cheng, I., et al., *Socioeconomic status and prostate cancer incidence and mortality rates among the diverse populations of California.* Cancer Causes and Control, 2009. **20**(8): p. 1431-1440.

13. Lyratzopoulos, G., et al., *Population based time trends and socioeconomic variation in use of radiotherapy and radical surgery for prostate cancer in a UK region: continuous survey.* BMJ, 2010. **340**.

14. Byers, T.E., et al., *The impact of socioeconomic status on survival after cancer in the United States.* Cancer, 2008. **113**(3): p. 582-591.

15. Schymura, M., et al., *Factors associated with initial treatment and survival for clinically localized prostate cancer: results from the CDC-NPCR Patterns of Care Study (PoC1).* BMC Cancer, 2010. **10**(1): p. 152.

16. Marlow, N., et al., *Disparities Associated with Advanced Prostate Cancer Stage at Diagnosis.* Journal of Health Care for the Poor and Underserved, 2010. **21.1**: p. 112-131.
